# National strategies for knowledge translation in health policy-making: A scoping review of grey literature

**DOI:** 10.1186/s12961-023-01089-0

**Published:** 2024-04-20

**Authors:** Balázs Babarczy, Julia Scarlett, Tarang Sharma, Péter Gaál, Balázs Szécsényi-Nagy, Tanja Kuchenmüller

**Affiliations:** 1 Unit of Knowledge Management, Evidence and Research for Policy-Making, Division of Information, Evidence, Research and Innovation, World Health Organization Regional Office for Europe, UN City, Marmorvej 51, 2100 Copenhagen Ø, Denmark; 2 Present Address: Syreon Research Institute, Budapest, Hungary; 3 Present Address: Evidence to Policy, Gyvelvej, Hoersholm, Denmark; 4 Health Services Management Training Centre, Semmelweis University Health Services Management Training Centre, Kútvölgyi Út 2, Budapest, 1125 Hungary

**Keywords:** Knowledge translation, Evidence-informed policy-making, Health planning, EVIPNet Europe

## Abstract

**Background and objectives:**

Without strategic actions in its support, the translation of scientific research evidence into health policy is often absent or delayed. This review systematically maps and assesses national-level strategic documents in the field of knowledge translation (KT) for health policy, and develops a practical template that can support Evidence-informed Policy Network (EVIPNet) Europe countries in producing national strategies for evidence-informed policy-making.

**Methods:**

Websites of organizations with strategic responsibilities in KT were electronically searched, on the basis of pre-defined criteria, in July–August 2017, and an updated search was carried out in April–June 2021. We included national strategies or elements of national strategies that dealt with KT activities, as well as similar strategies of individual institutions with a national policy focus. Two reviewers screened the strategies for inclusion. Data were analysed using qualitative content analysis.

**Results:**

A total of 65 unique documents were identified, of which 17 were eligible and analysed for their structure and content. Of the 17, 1 document was a national health KT action plan and 6 documents were institution-level KT strategies. The remaining 10 strategies, which were also included were 2 national health strategies, 5 national health research strategies and 3 national KT strategies (not specific to the field of health alone). In all, 13 structural elements and 7 major themes of health policy KT strategies were identified from the included documents.

**Conclusion:**

KT in health policy, as emerged from the national strategies that our mapping identified, is based on the production and accessibility of policy-relevant research, its packaging for policy-making and the activities related to knowledge exchange. KT strategies may play different roles in the complex and context-specific process of policy-making. Our findings show that the main ideas of health-specific evidence-informed policy literature appear in these strategies, but their effectiveness depends on the way stakeholders use them. Specific knowledge-brokering institutions and organizational capacity, advocacy about the use of evidence, and close collaboration and co-decision-making with key stakeholders are essential in furthering the policy uptake of research results.

**Supplementary Information:**

The online version contains supplementary material available at 10.1186/s12961-023-01089-0.

## Contributions to the literature

- Research has shown that the policy uptake of scientific findings needs to be reinforced through targeted, strategic interventions, which include the development of knowledge translation (KT) strategies.
- We have mapped a set of relevant national KT strategies in health policy, and identified their common structural elements, as well as the KT mechanisms.
- We have found only a few KT strategies in health policy, which may be one of the explanations for the slow adoption of research and evidence into health policy.
- On the basis of the analysis of the identified documents, we propose a practical template which could, with adaptations as necessary to the local context, serve as a benchmark for developing national KT strategies.
- Compiling and analysing information from grey literature should facilitate learnings that are often missing within the scientific literature on KT, and we hope this supports peer learning amongst countries.
- Such a template can facilitate development in this area, but written KT strategies are only a small technical part of an effective KT process.

## Background

Despite efforts to put research evidence at the forefront of health policy-making, a gap between evidence and policy remains [1]. Knowledge translation (KT) is an approach to promote the use of research evidence in policy and practice [2, 3]. While the term “KT” has been most prominently used in healthcare and policy settings, there is a wide variety of terms used across disciplines to describe evidence-uptake activities, all of which can be assimilated to a larger spectrum of similar activities, designated by Estabrooks et al. [4] as knowledge utilization.

To ensure that national health policy-making is routinely informed by the best available, context-sensitive research evidence, the WHO launched Evidence-informed Policy Network (EVIPNet). EVIPNet Europe promotes among its member countries the use of a range of KT products, such as the development of evidence briefs for policy (EBPs; summaries of policy-relevant evidence on a high-priority topic), and policy dialogues (multi-stakeholder discussions of evidence briefs used to complement scientific evidence with tacit knowledge). These approaches have proven to be effective in promoting KT and evidence-informed policy-making (EIP) in various case studies [5–7]. In this scoping review, we wanted to explore how those and other similar efforts are reflected in policy documents at the national level.

In 2013, the BRIDGE project published a systematic inventory of KT strategies in healthcare across Europe, with a primary focus on knowledge brokering organizations [8]. The current study focuses on the adoption of this technology based on the results of that project when defining the scope of its search for KT strategies.

The objective of this article is to identify existing health-policy-specific KT strategies, analyse a systematically included sample of national KT strategies, assess their structure and content and look at how they strive to catalyse KT in their respective jurisdictions. Firstly, a scoping review can provide a snapshot of a structured approach to setting of KT strategies among the countries. Secondly, it may add to our understanding of how KT is envisaged by actors who are dedicated to its implementation. Thirdly, such an analysis offers the possibility to produce a strategy template which may be used, among other sources, as a benchmark for the development of national KT strategies in EVIPNet Europe countries, and other countries wishing to strengthen EIP by the adoption of a country-specific strategy.

## Methods

This qualitative study is a structured and systematic mapping of national strategies for KT, collected from the websites of pre-selected KT-related institutions globally, between 3 July and 21 August 2017 (searches carried out by JS and BB). The search was updated between 21 April and 30 June 2021 (searches carried out by BSzN). The update of the search was complemented by e-mails sent to relevant institutions in the countries included in our review, asking for any new KT strategies or updates of earlier ones. Grey literature was chosen as the focus of the study because we wanted to examine the real-world reflection of KT concepts and methods; therefore, a review of actual national or sub-national KT strategies appeared to be the most appropriate strategy.

As the study is based on grey literature, it was not possible to follow a usual systematic review methodology. It is, however, systematic in its search of strategic documents, and in the extraction process of their structure and content (see below and in Additional file 2). First, we identified a pool of published KT strategies, and selected relevant documents on the basis of pre-defined inclusion and exclusion criteria (see below). Second, qualitative content analysis was carried out on the documents included in the study, and the relevant data were extracted, on the basis of which we elaborated a template with key elements for the development of KT strategies in health (for this output, see Additional file 2).

### Identification of relevant KT strategies

The identification process had four main steps. First, we defined the scope of countries and institutions to consider. We included EVIPNet countries, plus countries whose one or more institutions were included in the BRIDGE report [8]. Second, the types of institutions potentially relevant for KT were identified, based on the BRIDGE report’s findings and an earlier mapping of health research strategies undertaken by WHO/Europe (Additional file 1). The websites of the following institutions from the selected countries were screened: Ministries of Health, Ministries of Social Affairs, Ministries of the Economy/Employment, Ministries of Education/Culture, Ministries of Science/Research, the Government/Prime Minister’s Office/Presidency, Academy of Sciences/Council for Science and Technology and public health agencies/institutes.

Third, we created an initial pool of KT strategies by searching the websites of the selected institutions in three languages: English, Spanish and French (due to skills of the author team). All documents were searched within the websites via Google, using the following keywords: (“knowledge translation” OR “knowledge transfer”) AND “strategy”. This simple search strategy was designed to increase the specificity, without tipping the balance with its sensitivity, due to the scoping nature of this review (that is, avoid the inclusion of too many irrelevant documents).

The search keywords “transfert de connaissances”, “application des connaissances”, “échange de connaissances” and “echange de connaissances” were added in countries where French is an official language, on the basis of Lacouture et al. [9], while the search keywords “transferencia de conocimientos” were added in countries where Spanish is an official language, on the basis of Potau et al. [10].

Additional documents were added through hand searching to complement the formal systematic search, on the basis of the expert advice of one author (TS). The search process was updated in 2021.

The documents were assessed for inclusion by one researcher (BB), according to the pre-defined inclusion and exclusion criteria (Table 1). A second researcher (JS) also checked both the included and the excluded documents against the same criteria. The final list of included strategies was elaborated through consensus.

**Table 1:** Inclusion and exclusion criteria

Inclusion	Exclusion
National knowledge translation (KT) strategies for policy in health	National health strategies with no or very limited information on KT
National strategies on knowledge translation or knowledge transfer in general, even if it is not specific to health or policy	National research strategies that only mention KT, and not in a policy context
National health strategies or national research strategies if they contain substantial elements on KT to health policy (that is, specific objectives or actions, not only a mention of KT as an objective itself)	All documents with a format other than a national strategy, for example: • Supra-national guidance documents about the KT process • Documents on KT-related activities, for example, health technology assessment (HTA), other than national strategies • Strategies of individual institutions if they do not contain substantial elements on how to inform national health policy • Documents (mostly short in size) that do not resemble strategies, for example, newsletters or HR guidance documents • Reviews, evaluations and models serving as background documents for strategy development work
Strategies of individual institutions if they contain substantial elements on how to inform national health policy (that is, the institution has a national-level mandate and/or importance)	

The inclusion criteria were: national KT strategies for policy in health, national health strategies or national research strategies if they contain substantial elements on KT to health policy (that is, specific objectives or actions, not only a mention of KT as an objective itself) and strategies of individual institutions if they contain substantial elements on how to inform national health policy (that is, the institution has a national-level mandate and/or importance). National strategies on knowledge translation or knowledge transfer in general, even if they are not specific to health or policy, were also considered for their structure. We did not strictly exclude KT-related strategic documents from non-health disciplines, but we only considered documents where the aim was in line with the definition below.

We defined KT-related strategic documents as those that conformed with WHO’s following definition of KT:

….the exchange, synthesis, and effective communication of reliable and relevant research results. The focus is on promoting interaction among the producers and users of research, removing the barriers to research use, and tailoring information to different target audiences so that effective interventions are used more widely.[11]

The exclusion criteria were: national health strategies with no or very limited information on KT; national research strategies that only mention KT, and not in a policy context; and all documents with a format other than a national strategy. (For details, see Table 1)

### Framework for data extraction and analysis

Both headings and content elements were extracted and clustered. (For the extraction table, see Additional file 3.) Conventional content analysis, as outlined by Hsieh and Shannon [12], was used to analyse the extracted elements, and was undertaken by one researcher (BB). During extraction, themes were identified until saturation was reached; that is, adding new documents did not produce any new themes.

These elements were organized into themes as follows: headings were regrouped under themes of structure, and the KT-relevant content of the strategies was used to identify themes of strategic objectives and actions.

The resulting template (Additional file 2) presents the main elements of national strategies on KT, identified via a content analysis. Its aim is to present a review of existing practices that may be used as a benchmark in the development of national KT strategies to be produced in EVIPNet Europe countries. For details of the analysis process, see the data extraction output in Additional file 3.

The PRISMA checklist for scoping reviews [13], applicable to this article, is available in Additional file 4. The review protocol was not registered in the public domain, only in the WHO Regional Office for Europe records.

## Results

In this section we first present the number and type of KT documents identified through the systematic search strategy. Then, we present the findings of the content analysis that were used to develop our template in Additional file 2.

### KT strategies identified

**Fig. 1:**
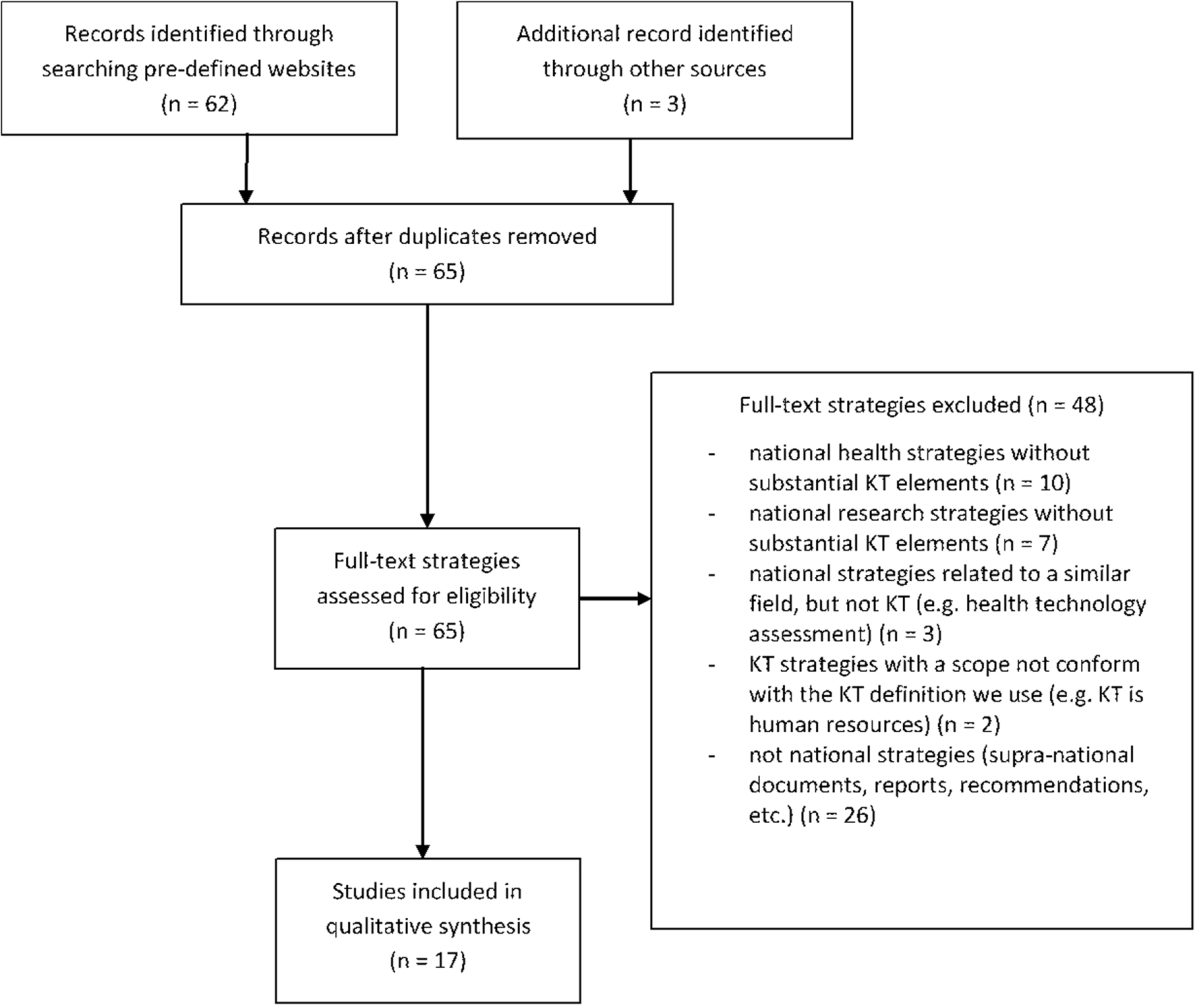
PRISMA flowchart of the screening process

A total of 62 documents were identified through the website searches, and 1 additional document [14] was added through hand searching. Later, the more recent versions of two strategies were also included [15, 16], but both earlier and later versions of these two strategies were included in the analysis. How the documents were processed is illustrated on Fig. 1 below.

On the basis of the criteria in Table 1 above, 17 documents were included in the study:

- 1 national KT action plan developed by EVIPNet [17],
- 2 national health strategies with substantial elements on KT (that is, specific objectives or actions, not only a mention of KT as an objective itself) [18, 19],
- 5 national health research strategies with substantial elements on KT [20–24]
- 3 national KT strategies, though not specific to health or policy [25–27]
- 6 institution-level KT strategies with a national importance [14–16, 28–30].

Out of the six institutional-level strategies, five were made by national research or KT institutes, and one by a university in a small country. The three national KT strategies that were not specific to health were used to identify themes related to structure, but their content was not included in the template as deemed not relevant.

The included documents exhibit a wide geographical variety: two were from the Middle East [19, 23], five from the Americas [14, 15, 25, 27, 29], three from Sub-Saharan Africa [16, 17, 30], six from Europe [18, 20–22, 24, 28] and one from Asia [26].

### The structure and content of KT strategies

The KT strategy template in Additional file 2 was developed on the basis of the 17 national strategies identified above. It presents the possible building blocks that can be used for the development of national KT strategies. It gives a list of headings for the strategic document (which we hereafter refer to as structure), and examples of what may be discussed under those headings (content).

In terms of their structure, the reviewed strategies consisted of three main parts. First, an introductory section comprising different elements (headings), including some of the following: foreword by a decision-maker responsible for the institution producing the strategy, executive summary, a definition of KT and of the strategy’s scope, vision and/or mission statement, elements of context (for example, country description, earlier KT efforts, other documents relevant in the area of KT, mandate of the institution producing the KT strategy or theoretical foundations), situation analysis and the goals, actors and processes of strategy development.

Following the introduction, the strategic objectives and actions related to KT were set out, often in a table format with indicators and responsible organizations. (For its details, see below.) Often in the closing segment, there was usually a section on implementation, measurement and evaluation, and in some cases also one dedicated to the development of research on KT itself. Finally, annexes contained several elements of content, from the list of stakeholders involved in strategy development to references and glossaries.

The above categories are not mutually exclusive; that is, some elements that have a separate heading in one strategy may figure below another heading in a different document (for example, vision can be part of the foreword). For the number of strategies containing each type of headings, see Table 2. For details of the structural elements, see Additional file 2.

**Table 2:** Number of different strategies in our sample, containing given elements as explicit and distinct sections

Section	Number of strategies containing the section
Foreword	4
Executive summary	4
Definition(s) and scope	6
Vision and mission	7
Context	10
Situation analysis	7
Justification for producing the strategy	2
Actors and processes of strategy development	5
Strategic objectives and actions	17 (for details, see Table 3)
Implementation	8
Measuring and evaluation	13
KT research	3
Annexes	7

In terms of strategic objectives and actions, seven main themes emerged using the conventional content analysis method as described by Hsieh and Shannon [12]. (For the list of main themes, see Table 3. For details about each theme, see Additional files 2 and 3)

**Table 3:** Number of different strategies in our sample dealing with content items on strategic objectives and actions

Content item	Number of strategies containing the item
Creation of policy-relevant research	8
Assuring the accessibility of research publications	5
Knowledge exchange between researchers and other stakeholders	10
Capacity-building for KT	12
Institutions responsible for KT	12
Cooperation among different actors	12
Communication (advocacy) about research and KT	10

Most KT strategies focused on the production of policy-relevant knowledge, and how to integrate EIP processes. The first step of this EIP process, as constructed from the strategies identified, is to encourage the production of research with policy relevance, and to collect its findings in easily accessible repositories. This is followed by knowledge exchange processes, within which stakeholders meet at dedicated fora, or produce written documents that are easily understood and used by the decision-makers (packaging knowledge), thus transmitting the essence of research findings towards practice. This process is underpinned in most strategies by the establishment or designation of appropriate institutions acting as knowledge brokers, capacity-building within those institutions and among different stakeholders, cooperation of different institutions with a similar role and communication (advocacy) around KT to capture the interest and involvement of all potential stakeholders.

Because not all documents’ entire content is relevant or applicable in accordance with our KT definition, only the elements referring to KT with the same lens as our definition, were retained for the template. The phrases in the template are, for the most part, not exact citations, but have been slightly reformulated. Where the reformulation is more than purely grammatical, it is signalled by text [in brackets]. See Additional file 2.

## Discussion

This paper aims to contribute to the KT discipline by the systematic screening and analysis of KT strategies in the area of health policy (including public health). The results of this study in terms of the number and types of KT strategies identified, the identification of their common key topics and the synthesis of their structure and content into a practical template contribute to a better understanding of the area. A focus on health policy means that we were potentially missing important aspects of KT in general, but may be justified by the sector’s specific role within the realm of KT efforts, as described by Cairney [31].

The elements of the strategies that we identified largely reflect the main directions of KT as described in the evidence-informed health policy literature. For instance, Lavis et al. [32] distinguished between seven key strategic domains of KT: general climate, production of research, “push” efforts by researchers towards the users of research, efforts to facilitate user pull, user pull efforts themselves, exchange efforts and evaluation. Most elements identified by our review can be linked to some of these domains. In this study, we found no significant difference between one national KT strategy specifically produced using the framework of an EVIPNet programme [17] – which is indeed the only national KT strategy per se – and the other documents, suggesting a common understanding of KT objectives and actions between different national and international actors.

While “general climate” is a concept that lends itself with relative difficulty to practical implementation in KT strategies, most of the elements we found in the vision and mission chapters of national KT strategies may be assimilated to that topic. (For details, see Additional file 2.) “Production of research”, on the contrary, is quite explicitly mirrored by our KT strategy item of creating policy-relevant research.

“Push efforts” are most clearly represented by the strategic objectives we identified under the topic of communication (advocacy) about research. “Efforts to facilitate user pull” correspond in many ways to the elements we regrouped under the heading of assuring the accessibility of research, while “user pull efforts” are partly covered under institutions and partly under capacity-building. (The latter are also transversal and cover several topics of Lavis et al. [32] framework. Finally, “exchange efforts” are to be found within our knowledge exchange topic, while “evaluation” is situated under measurement and evaluation.

Some KT mechanisms, present in the literature, were missing from the strategies we reviewed. Therefore, the template presented in Additional file 2 would probably need to be complemented by some of those, based on further research and consideration.

This demonstrates a relatively clear echo of the evidence-informed health policy school in the national documents that we found. The fundamental question remains: how can these strategies, often initiated by either members of the research community or, in developing countries, by donors interested in making effective policy change through the operational use of evidence, influence the political process of policy-making?

Berman et al. [33] present a successful example of institutional capacity-building within an EVIPNet knowledge translation platform in Africa. The main objectives of KT strategies identified there, namely the production of policy-relevant research, its transformation via documents such as systematic reviews and EBPs, and knowledge brokering activities, are in line with our findings. The WHO guide for the establishment of health observatories in Africa [34] also identifies very similar missions for health observatories, suggesting that they may act as knowledge brokers in contexts where they are operational.

Other studies tend to focus on the details of certain push, user pull or knowledge exchange strategies. Sarkies et al. [35] emphasize the effectiveness of written summaries for the purposes of KT. They also underline the importance for researchers or knowledge brokers to be involved in agenda setting, building trust and a shared vision with policy-makers, underpinning change mechanisms, while keeping in mind the importance of communication strategies and of catering for the resource needs of changes incepted through KT.

Cooperation between different actors is an important objective, as underlined by the successful KT strategies documented by Jessani et al. [36], which show that the identification of policy needs right from the beginning of the research or research synthesis process is a key approach. Uneke et al. [37], McDonald and Viehbeck [38], Ferdinand et al. [39] and Moore et al. [40] also provide examples of cooperation among stakeholders of the KT process, including the common definition of objectives by researchers and policy-makers, often involving knowledge brokers.

In terms of strategies to increase the efficacy of KT mechanisms, Bauer et al. [41] identify the key attributes of KT efforts of research organizations active in the field of climate change, where the impact of scientific findings on policy agenda has recently been significant. They distinguish three goals of KT organizations: salience (policy-relevance), credibility (scientific soundness) and legitimacy (transparency and institutional competence), which are assured by organizational, procedural and rhetorical arrangements. These, again, can be directly linked to the capacity-building, institutions, cooperation and communication (advocacy), elements, from the strategies we identified.

Meanwhile, Malama et al. [42], for example, report on shortcomings of KT in Zambia, including why it happens, and which factors decide whether KT strategies stay on paper, or have actual impact. From a political science point of view, Cairney [43, 44] argues that the process of policy-making is not linear, as many supporters of EIP would like to see it. He demonstrates that several governments are simply not in a position to oversee the introduction and implementation of an evidence-informed policy initiative, as they are confronted with a multitude of political actors and stakeholders in decentred policy arenas. Decisions, in this sense, are made in a complex web of decision venues, as the sum of many interests, and no actor – not even the central government – has complete information, understanding and capacity to implement their decisions.

Hukkinen [45] presented an interesting model of EIP within such a dispersed arena. He shows that a model based on Baumgartner and Jones’s [46] punctuated equilibrium framework may explain the dynamics of knowledge brokering within the European Commission’s deliberations on sustainability research policy. But punctuated equilibrium is not the only policy change framework that can be applied for the description of evidence-driven reforms. In an earlier contribution, Babarczy and Imre [47] applied Sabatier and Jenkins-Smith’s advocacy coalition framework [48] to explain an overarching health financing reform in Hungary. According to both frameworks, considering the complexity of the decision-making process, the role of policy brokering – that is, the need to “cross-fertilize” different coalitions and decision venues with evidence-informed solutions – is key.

Indeed, Cairney [31] presents seven political science theories that may explain policy change – multiple streams, punctuated equilibrium, social construction, the narrative policy framework, the advocacy coalition framework, policy transfer and complex systems, many of which describe the policy formation and implementation process as one that may take a long time – and efforts may sometimes seem futile – in contexts where decision-making is not necessarily linear. On the other hand, a large part of these theories emphasize the importance of images and perceptions in framing policy. While Parkhurst [49] clearly presents the possible bias of policies with claims of being evidence-based or evidence-informed, this perception may help their implementation at some point of the complex policy process. Therefore, we may see the strategic documents analysed here as potential reference points, embedded in the terminology and theoretic framework of the EIP in health policy movement, which can support the implementation of future policy interventions.

One should also not forget that KT may not only concern democratic states, on which most policy science theories focus. For example, Dye [50] presents a case of electrification policy in Tanzania, where different phases of authoritarian rule led to different styles of decision-making. While President Kikwete’s term (2005–2015), characterized by competing interest groups, impeded the effective implementation of policies, President Magufuli (2015–2021) was able to establish genuinely centralized rule. This reminds us that the decentred character of multiple policy venues is not consubstantial to the policy process: there may well exist regimes where unitary decision-making is actually possible, and KT may be an even stronger reference point and source of legitimacy in such circumstances. In those instances having a strong national KT strategy for health-policy may be most effective and may lead to implementation.

In summary, KT is a complex and highly context-specific process, which is by no means only a simple technical issue. Technocratic coordination tools, such as written KT strategies can support EIP, but may also be a referential support of policies that some stakeholder would like to implement for a completely different reason. To be really effective, KT efforts should pay attention to the values and interests present in the decision-making process and minimize the bias that can result from a narrow focus on a single source of evidence.

### Strengths and limitations

The methodological strengths of this study include its wide-ranging sample in terms of geographic areas and types of institutions, and of its practical output. As this scoping review focuses on grey literature, these learnings on their own remain hidden and not easily accessible by countries. The template developed upon the content and structure of national KT strategies we identified (Additional file 2) may help EVIPNet Europe member countries, as well as other entities working in KT, in developing their strategic thinking, and provide a basis for initiating or strengthening EIP in their contexts. Filling in the different parts with elements applicable to the local context, alongside consideration for theoretically embedded frameworks such as Lavis et al. [32], should assist in identifying priorities and ways forward in strategic actions for KT.

Principal limitations of this study relate to its basis in its scoping nature and also being limited to only grey literature, which does not permit the scientific rigour of a systematic review, nor the possibility for critical appraisal of the quality of the included documents. Furthermore, our search covered only the health sector, and a specific set of institutions, which excluded other governmental sectors and institutions, and their KT strategies. While this decision regarding the scope of the research obviously limited the number of identified documents, we are convinced that health is an important area of public policy, to be studied alone.

Another limitation is that, although two researchers assessed the strategies for inclusion, the analysis was only conducted by one researcher who undertook the coding of the different concepts and themes alone. Additionally, only a set of limited websites were searched and had a limited set of search terms as a mapping exercise to increase specificity; therefore, selection bias is inherent. Language restrictions also limited our potential scope of findings, although we included three wide-spread world languages. However, we feel we countered this by having a wide net in terms of geographical distribution and types of institutions targeted. Overall, we do feel that the findings of this study contribute to the literature in this field and provide a practical building block for the development of KT strategies. Having such implementable and actionable templates are often lacking in the published scientific literature, and therefore, we believe that this study facilitates peer learning across countries in EIP.

### Future research needs

Currently, we know little about the extent to which strategic objectives are turned into concrete political action. It would therefore be important to understand what is needed from different stakeholders to make KT a reality, and also what is needed from national and regional stakeholders (for example, WHO/Europe) to make KT strategy development a political priority and what is needed then for its implementation.

Monitoring and evaluation is embedded in most strategic frameworks. It should include reflections on the way that strategy development contributes to action in different jurisdictions and political contexts. Research is needed to evaluate which strategic actions work, and also how being enshrined in an official document supports their implementation, much like the research Ongolo-Zogo et al. [51] performed with regards to KT platforms.

## Conclusion

Despite the growing importance of KT within the health policy literature and a number of successful examples presented, there is currently no synthesis of national strategies in this area. The objective of this article was to identify existing national KT strategies, assess their structure and content, reflect upon their objectives in the context of scholarship on policy-making and develop a practical template on that basis, which can be used by country teams embarking on KT strategy development. The main content elements of KT strategies, that is, the production and accessibility of policy-relevant research, the activities related to knowledge synthesis and brokering, cooperation and communication, and the institutions and capacity-building required, largely reflect the main trends found in recent KT literature. However, research is still needed to evaluate their effectiveness in actually shaping public policy. The proposed template is only a guide which may need to be further complemented, and also adapted according to the individual needs of countries embarking upon EIP strengthening.

### Supplementary Information

**Additional file 1. **Mapping of available national health research strategies (NHRS). Output of previous research indicating types of organizations potentially involved in KT.

**Additional file 2. **Knowledge translation (KT) strategy template. Guidance for the development of KT strategies, based on research in this article.

**Additional file 3. **Extraction table of KT strategy elements. Populated extraction table of structure and content elements of the identified KT strategies.

**Additional file 4.** PRISMA checklist for scoping reviews. Checklist of reporting standards.

## Data Availability

The datasets supporting the conclusions of this article are included within the article and its additional files.

## Abbreviations

BRIDGE: Brokering Knowledge and Research Information to Support the Development and Governance of Health Systems in Europe
EBP: Evidence brief for policy
EIP: Evidence-informed policy-making
EVIPNet: Evidence-informed Policy Network
KT: Knowledge translation
KTP: Knowledge translation platform
