# Supplementary material for: National strategies for knowledge translation in health policy-making: A scoping review of grey literature
Source: Health Res Policy Syst. 2024 Apr 20;22:50. doi: 10.1186/s12961-023-01089-0 (PMC11031914; doi:10.1186/s12961-023-01089-0)
Supplement: Supplementary file 1 — Additional file 1. Mapping of available national health research strategies (NHRS). Output of previous research indicating types of organizations potentially involved in KT. [file 12961_2023_1089_MOESM1_ESM.docx]

## Additional file 1: Mapping of available national health research strategies (NHRS)

| **Country** | **Title of the strategy** | **Year** | **Level** | **Owner of the strategy** | **Produced by** |
| --- | --- | --- | --- | --- | --- |
|  |  |  |  |  |  |
|  |  |  | ***1*** *- National*  ***2*** *-Subnational* ***3*** *- Institutional  4 - Disease-specific* |  |  |
| **Russia** | Стратегия развития медицинской науки в Российской Федерации /Medical research development strategy in the Russian Federation | 2013 - 2025 | 1 | The Russian Federation | Ministry of Health of the Russian Government |
| **Norway** | The Health and Care 21 strategy | 2014 - | 1 | Ministry of Health and Care Services | The Ministry of Health and Care Services |
| **Iceland** | Act on Scientific Research in the Health Sector | 2014 - | 1 | Ministry of Welfare | Ministry of Welfare |
| **Croatia** | Strategic Research Agenda: Health Research  Priorities for Croatia | 2009 - 2013 | 1 | Coordination of Research Policies with the Western Balkan Countries | School of Medicine, University of Split - consortium |
| **Estonia** | Research Development and Innovation Strategy  for the Estonian Health System | 2015 - 2020 | 1 | Ministry of Social Affairs | Estonian Academy of Sciences and its Standing Committee on Medical Science |
| **Finland** | Health Sector Growth Strategy for Research and  Innovation Activities. Roadmap | 2016 - 2018 | 1 | Ministry of Social Affairs and Health, Ministry of Education and Culture, Finnish Funding Agency for Innovation, Academy of Finland | Ministry of Employment and the Economy, Ministry of Social Affairs and Health, and Ministry of Education and Culture |
| **Germany** | Health Research Framework Programme of the Federal Government | 2010 - | 1 | Federal Ministry of Education and Research | Federal Ministry of Education and Research (BMBF) Health Research Division |
| **Germany** | Roadmap for the German Health Research  Program of the Federal Government | 2007 - | 1 | Federal Ministry of Education and Research | German Health Research Council (GFR) of the Federal Ministry of Education and Research |
| **Ireland** | A Strategy for Health Research –  making knowledge work for health | 2001 - | 1 | Department of Health and Children | Department of Health and Children, the Government of Ireland |
| **Malta** | National Strategy for Health Research and Innovation | 2011 - | 1 | The Malta Council for Science and Technology | Malta Council for Science and Technology |
| \|  \| \| --- \| |  |  |  |  |  |
| **England** | Best Research for Best Health. A new national health research strategy | 2006 - 2010 | 2 | Research and Development Directorate, Department of Health | Research and Development Directorate, Department of Health |
| **Scotland** | Delivering Innovation through research – Scottish  Government Health and Social care Research Strategy | 2015 - | 2 | Chief Scientist Office | Chief Scientist Office |
| **Wales** | Research Strategy Public Health Wales | 2015 - 2018 | 2 | Public Health Wales | Policy, Research and International Development Directorate, Public Health Wales |
| **Northern Ireland** | Research for Better Health & Social Care.  A Strategy for Health & Social Care Research and Development in Northern Ireland | 2016 - 2025 | 2 | Department of Health, Social Services and Public Safety | Department of Health, Social Services and Public Safety |
| **Ireland** | National Health Research Board Strategy. Research. Evidence. Action. | 2016 - 2020 | 3 | Health Research Board, Ireland | Health Research Board, Ireland |
| **Sweden** | The Swedish Research Council’s Research Strategy | 2009 – 2012 | 3 | Swedish Research Council | Swedish Research Council |
| **UK** | Medical research council strategic plan. Research changes lives. | 2014 - 2019 | 3 | Medical Research Council, UK | Medical Research Council, UK |
| **Ireland** | Mental Health Commission Research Strategy | 2005 - | 3,4 | Mental Health Commission | Mental Health Commission |
| **UK** | Mental Health Foundation Research Strategy | 2016 - 2020 | 3,4 | Mental Health Foundation | Mental Health Foundation |
| **Denmark** | Mental Health Centre for Child and Adolescent Psychiatry Capital Region of Denmark. Research Strategy 2013 | 2013 - | 3,4 | Mental Health Centre for Child and Adolescent Psychiatry Capital Region of Denmark | Mental Health Centre for Child and Adolescent Psychiatry Capital Region of Denmark |
