# Supplementary material for: National strategies for knowledge translation in health policy-making: A scoping review of grey literature
Source: Health Res Policy Syst. 2024 Apr 20;22:50. doi: 10.1186/s12961-023-01089-0 (PMC11031914; doi:10.1186/s12961-023-01089-0)
Supplement: Supplementary file 2 — Additional file 2. Knowledge translation (KT) strategy template. Guidance for the development of KT strategies, based on research in this article. [file 12961_2023_1089_MOESM2_ESM.docx]

## **Additional file 2: Knowledge translation (KT) strategy template**

This document presents the main elements of national strategies on knowledge translation (KT), identified via a content analysis. Its aim is to present a review of existing practices that may be used as a benchmark in the development of national KT strategies to be produced in EVIPNet Europe countries. Some KT mechanisms, present in the literature, were missing from the strategies we reviewed. This framework would probably need to be complemented by some of those, based on further research and consideration.

The mapping and search identified 17 eligible documents, the content and structural elements of which add up to form the present document (for details, see the extraction output in Annex 3). Both headings and phrases of text were extracted, in order to present a suggestion for the structure as well as the content of strategic documents.

Knowledge translation is understood here as the exchange, synthesis, and effective communication of reliable and relevant research results. The focus is on promoting interaction among the producers and users of research, removing the barriers to research use, and tailoring information to different target audiences so that effective interventions are used more widely (3). Because not all documents’ whole content is relevant or applicable in accordance with this definition*,* only the elements referring to KT in the sense of this were retained.

The phrases of what follows are, for the most part, not exact citations, but have been slightly reformulated. Where the reformulation is more than purely grammatical, it is signaled by text [in brackets].

The outline of a KT strategy may be the following. (Details for each part are given below.)

[*A)* *Introductory parts*](#_Toc4923145)

[Foreword](#_Toc4923146)

[Executive summary](#_Toc4923147)

[Definition and scope](#_Toc4923148)

[Vision and mission](#_Toc4923149)

[Context](#_Toc4923150)

[Situation analysis](#_Toc4923151)

[Justification for producing the document](#_Toc4923153)

[Actors and processes involved in the development of the document](#_Toc4923154)

[*B)* *Strategic objectives*](#_Toc4923155) *and actions*

[*1.* *The process of KT*](#_Toc4923156)

[Production of policy-relevant research](#_Toc4923157)

[Accessibility of publications](#_Toc4923158)

[Knowledge exchange](#_Toc4923159)

[*2.* *Factors facilitating KT*](#_Toc4923160)

[Capacity building for KT](#_Toc4923161)

[Institutions](#_Toc4923162)

[Cooperation](#_Toc4923163)

[Communication](#_Toc4923164) (advocacy)

[*C)* *Closing part*](#_Toc4923166)

[Implementation](#_Toc4923167)

[Measuring & evaluation](#_Toc4923168)

[KT research](#_Toc4923169)

[Annexes](#_Toc4923170)

[References](#_Toc4923172)

*Number of strategies containing each section:*

| **Section** | **Number of strategies containing the section** |
| --- | --- |
| Foreword | 4 |
| Executive summary | 4 |
| Definition(s) and scope | 6 |
| Vision and mission | 7 |
| Context | 10 |
| Situation analysis | 7 |
| Justification for producing the strategy | 2 |
| Actors and processes of strategy development | 5 |
| Strategic objectives and actions | 17 |
| Implementation | 8 |
| Measuring and evaluation | 13 |
| KT research | 3 |
| Annexes | 7 |

# *Introductory parts*

In the following, the main structural elements figuring at the beginning of KT strategies are listed, with a brief description of their usual content in the strategies analysed.

## Foreword

Four documents contain a section called Foreword (4, 6-8). These usually serve as an expression of high-level (political) commitment to the objectives and actions of the strategy. Examples of forewords are the following:

- Brief description of the institution by the chairman and the chief executive (4),
- Strategic vision by the head of the Strategy Steering Group and a key researcher (7),
- Foreword by the Minister of Health (6),
- Foreword by the Chairman of the institution’s Council (8).

## Executive summary

Four documents contain a summary at the beginning (4, 7, 8, 11). This gives an opportunity for the busy reader to briefly apprehend the main content of the strategy, and is therefore very useful. It is most often called Executive summary, but it may also be part of the section called Introduction.

## Definition and scope

Because knowledge translation is a relatively recent concept, with multiple possible interpretations, it is worth defining its scope at the beginning of the strategy. Many KT strategies contain a definition of scope (2, 4, 6-8, 12), and also of timeframe (13). Putting the definition in a box may be a user-friendly way of presentation (7).

**Box 1: Examples of definitions in the KT strategies reviewed**

- *Knowledge translation is the exchange, synthesis and ethically-sound application of knowledge – within a complex system of interactions among researchers and users – to accelerate the capture of the benefits of research through improved health, more effective services and products, and a strengthened health care system. (2)*
- *Research is an attempt to derive generalizable and/or transferrable new knowledge by addressing clearly defined questions with systematic, rigorous and repeatable methods. Translation is a process of adding value to robust evidence so that it may be used. Innovation is a process that brings together various novel ideas in a way that they have an impact (on public health). (4)*
- *The concept of knowledge transfer is an important aspect in the process of institutional development and sustainability, which is a process to disseminate the results of health research in an easily and understandable way to have the greatest impact on health policy makers, health care providers, and the public. (6)*
- *In systems centered in the public sector, such as the health system, innovation should be understood as a dual dynamic process: 1) innovation within the public sector, focusing on the development and renewal of the sector’s own internal activities, and 2) innovation through activities supporting innovation in the public sector (e.g. R&D support and procurements supporting innovation that influence the development of other participants in the system). (7)*

##

## Vision and mission

Most documents set out the vision and/or mission that they have in mind when preparing the KT strategy. Five documents contain an explicit section of Vision (7, 8, 10, 11, 14)^[[1]](#footnote-1)^, four include a statement called Mission (8, 10, 14, 15)^[[2]](#footnote-2)^, and two documents comprise a chapter of Strategic vision (5, 8). Vision is usually applied to describe the state of the world as it would be after the implementation of the strategy. At the same time, mission is a broad formulation of what the value added of the strategic activities would be.

**Box 2: Elements of vision and mission statements in the KT strategies reviewed**

- *To promote evidence-informed policy making, innovation and the public interest (1);*
- *To support [ministry] staff in accessing evidence and the views of citizens (1);*
- *To promote a wide-range diffusion and systematic utilization of research findings relevant to decision making and to the conception of policies, programs and projects in health (5);*
- *Research and innovation activities must become more strategic and have a clearer basis in governance (9);*
- *To drive forwards the provision and use of evidence for decision-making across the public health system (4);*
- *To encourage [research] projects to plan for impact and mobilize knowledge [for policy making] (10);*
- *To support a more targeted, impact oriented, multidisciplinary research agenda (10);*
- *To foster a greater appreciation [by policy makers] of the value and impact of research, innovation and knowledge transfer (10);*
- *To facilitate research which impacts on policy, practice, new products, services, ways of thinking, attitudes and behavior, which ultimately contribute to the improved well-being of society (10);*
- *To establish processes by which the results of research can be transferred, and thus exploited and used [by policy making] (13);*
- *[To boost] innovation supporting the renewal and development of the health system (7);*
- *To improve the performance of the health care system and the adoption of policies based on the results of studies, evidence and information by providing training and financial support and motivation to conduct research on health systems (6);*
- *To foster the conduct of well-regulated high quality research that is responsive to the priority health needs of the community (14);*
- *To recognize and promote the transfer of knowledge via the exchange of creative and innovative ideas, research findings, experiences and skills between [higher education], research organizations, government agencies and the wider community (15);*
- *To support excellent research, generate relevant knowledge and promote its application in policy and practice (16);*
- *To excel in the creation of new knowledge and to translate that knowledge from the research setting to real-world applications in order to improve the health of citizens, provide more effective health services and products and strengthen the health care system (2).*

## Context

*A total of ten documents present some elements of context in their introductory part (2, 4, 5, 7, 8, 12-16)^[[3]](#footnote-3)^. The content of these elements varies, including the following:*

- *Country description (5)*
- *Earlier efforts made in the field of KT and further development needs (7, 12, 14, 16)*
- *Other strategic documents or legal references governing the field (2, 4, 12-14, 16)*
- *Mandate of the organization producing the strategy (2, 4, 14)*
- *Theoretical and institutional framework of the strategy (7, 15)*

## Situation analysis

## A situation analysis is a very important piece of strategy making, as it indicates the point of departure and the main challenges to be addressed by the strategy. In this respect, it is the foundation of strategic objectives and actions. Seven documents contain a section on situation analysis (5, 7, 8, 12-15). Both the SWOT analysis (8, 15) and the problem tree (13) may be useful approaches for the situation analysis to be based on.

## Justification for producing the document

Two documents contain a separate section about why the strategy has been developed in the situation described above (5, 15), and one additional document includes such elements in its Introduction (7).

## Actors and processes involved in the development of the document

The process of strategy development and the list of stakeholders involved, especially if these indicate a wide-ranging, participative approach with all relevant parties (scientists, practitioners, patients, different ministries, etc.) on board, may substantially increase the legitimacy of the document. Three of the documents we reviewed list contributors (7, 13, 15), and two of them discuss the process of elaboration (5, 8). Some other strategies provide details on these elements in annexes (see at the end of the document).

# *Strategic objectives and actions*

Objectives are a key structuring elements of strategies, as actions, responsibilities, timelines, etc. all derive from them. In several strategies, objectives, actions, the responsible entity, timelines, costs and key performance indicators (KPIs) are given in an organized way. A table format, following the outline below, is often used, e.g. in (1, 2, 5, 9, 16, 17).

| Objective | Action | Responsible body(ies) | Timelines | Costs | KPIs |
| --- | --- | --- | --- | --- | --- |
| Objective 1 | Action 1.1 |  |  |  |  |
|  | Action 1.2 |  |  |  |  |
| Objective 2 | Action 2.1 |  |  |  |  |

Strategic objectives usually mean putting in place a variety of KT mechanisms. These can be classified in two categories: putting place the individual steps of the KT process itself, and creating the environment supporting it. These are presented in more detail below.

## *The process of KT*

Knowledge translation means one should have policy-relevant research findings at hand, those findings (and those produced elsewhere) should be available in easy-to-access repositories, and the actual knowledge exchange process should be institutionalized. Examples of strategic objectives relative to these areas are given in Boxes 3-5 below.

**Box 3: Examples of strategic objectives for the production of policy-relevant research**

Eight documents reviewed mention activities related to the production of research relevant for translation and use in policy (4, 6-8, 10, 11, 15, 16). Out of these, the following are conform to our definition of KT:

- *Undertake research that peer reviewers commend as being of the highest quality (4);*
- *Focus researchers on research questions relevant to the evidence needed for public health (4);*
- *Define, and periodically review, health research priorities (6, 15);*
- *Commission [and fund] research studies, as deemed appropriate or as requested by state bodies and other stakeholders (16);*
- *Prepare an applied research program based on the development needs of the health system, the goal of which is to support the evidence and knowledge base of health policy through research, evaluation, and other coordinated organization and funding (7);*
- *Provide support for new applied research projects that meet the needs of the healthcare system using integrated knowledge translation approaches (16);*
- *Manage existing initiatives in targeted and strategic health areas to optimize knowledge translation for policy and/or practice (16);*
- *Apply diverse research methodologies appropriate for the range and complexity of public health research questions and contribute to methodological development (4);*
- *Strategize for impact from the inception of research projects as opposed to waiting until the completion of the project’s research activities and hoping for impact (10);*
- *Promote integrated knowledge-translation approaches, including implementation research, in order to ensure optimal translation of findings into policy and/or practice (16);*
- *Promote the secondary analysis and deeper exploitation of existing data sources, in order to deliver high-quality, high-impact evidence for policy and/or practice (16);*
- *A targeted approach complements investigator-initiated research by ensuring that a portion of […] investments is deliberately directed towards health and health system research priorities that reflect the evolving health needs and expectations […] and capture emerging national and international scientific opportunities (11).*

##

**Box 4: Examples of strategic objectives for the accessibility of publications**

Five documents include elements on making research findings available for use in other settings (5, 8, 14, 16, 18). With regards to their main content elements, the following may be retained:

- *Make research findings immediately accessible for healthcare decision making, the conception of policies and programs (5);*
- *Trace research products and build an inventory (5);*
- *Promote Open Science (e.g. open access to scientific publications and open access to data), as prerequisite for successful knowledge transfer policies (18);*
- *Strengthen and maintain a National Health Research Database (14);*
- *Launch a website/page dedicated to evidence for policy making (14);*
- *Provide access to [national library of research documents] to facilitate the use of research-based knowledge in decision-making (16);*
- *Health Research Repository developed and functional (8);*
- *Health research database maintained and strengthened (8);*
- *Information acquired and disseminated through digital libraries and e-systems (8).*

**Box 5: Examples of strategic objectives about the actual process of knowledge exchange**

A total of ten documents contain elements on efforts to achieve the take-up of relevant knowledge (1, 4, 6, 8-10, 12-14, 16). The following examples may be retained:

- *Manage internal and external research programs aligned with departmental priorities and act as a broker to harness learning and insights from the research community (national and international) to inform the work of the [administration] on key issues (1);*
- *Ensure that research outcomes and new innovations are implemented in [healthcare] services, and that the effects thereof are evaluated (9);*
- *Facilitate processes of dialogue between different social groups in the country on public policies in strategic areas, promoting their participation in the management of these (12);*
- *Support the robust evaluation of public health interventions including close interaction with partner organizations so as to contribute to effective decision-making by providers of public health services (4);*
- *Value and evaluate evidence from diverse sources and use it systematically to improve public health (4);*
- *Hold training workshops and/or conferences to share key findings [of research projects], so as to facilitate knowledge uptake (10);*
- *Hold meetings, seminars, workshops and conferences [on research outcomes relevant for policy] (6, 13, 16);*
- *Issue [policy-relevant research] bulletins and quarterly/annual reports (6, 16);*
- *Provide summaries of research studies (6);*
- *Develop and introduce an evidence review service for health service planners (16);*
- *Share research summaries and policy briefs developed and shared with stakeholders (8);*
- *Share research findings through multi-media approaches (8);*
- *Establish and hold policy dialogues (8);*
- *[Make] advocacy for utilization of health research findings and products strengthened (8).*

## *Factors facilitating KT*

Knowledge translation usually does not happen on its own: certain supporting conditions need to be present for it to actually take shape. Strategic objectives for the creation of these conditions may be split into four categories: (human) capacity building, institutions, cooperation among different institutions and communication (advocacy) to assure a wide uptake of research findings. Boxes 6-9 present examples for these, taken from the strategies reviewed.

**Box 6: Strategic objectives about capacity building for KT**

Twelve documents contain elements associated with (physical or human) capacity building for the better use of evidence (2, 4-6, 8, 9, 11-16). These include the following:

- *Invest in, deploy and share sophisticated research facilities, equipment and expertise (4);*
- *[Give] strong support for [public health research institutions] to build research capacity, capability and excellence (4);*
- *[Train] research communities to have a better understanding of the [health] services and their needs (9);*
- *Strengthen researchers/policy makers capacity in use of acquisition, assessment, adaptation and application of research evidence (14);*
- *[Promote] an improved knowledge and culture for research in [health] services (9);*
- *Reinforce different actors' capacity to better use research findings in decision making (5);*
- *Conduct trainings to mass media editors and journalists on reporting of research findings for public consumption (5, 14);*
- *Organize study trips [in countries with well-established KT practices] (5);*
- *Hold training workshops in the field of research and statistical analysis (6);*
- *Conduct training on systematic reviews of important research findings for policy development (14);*
- *Train district and regional health personnel on operational research (14);*
- *Conduct research translation workshops to program managers and practitioners (14);*
- *Conduct seminar for Members of Parliament on major health problems (14);*
- *Send staff on training courses internal and external (6);*
- *Promote national traineeships/internships, courses and diploma [in KT] (13);*
- *[Organize] virtual conferences in [KT] (13);*
- *[Grant] accreditation to professionals [in knowledge transfer] (13);*
- *[Offer] specialized advisory services in the elaboration of institutional policies [of KT] (13);*
- *Strengthen the curriculum of [higher education] based on industry and community [needs] (15);*
- *Promote access to the Cochrane Library, and support the training of individuals and groups to conduct high-quality and timely Cochrane Reviews, in order to inform healthcare decision-making (16);*
- *Strengthen the capacity of journalists in reporting health research findings (8);*
- *Strengthen the capacity of researchers on translation and packaging of research findings (8).*

**Box 7: Examples of strategic objectives about institutions**

Twelve documents reviewed contain references to the creation and work of institutions for KT (1, 2, 5-9, 12-15, 18). These include the following:

- *Put in place or reinforce exchange mechanisms between decision makers, researchers and civil society (5);*
- *[Create a] Research Services Unit within [public administration] that will ensure that research and evaluation is at the core of policy formulation and decision making (1);*
- *Appoint specialist leaders (6, 7), and set up a secretariat responsible for KT (5);*
- *Form a team of health systems research in the Ministry of Health (6);*
- *Establish [territorial] knowledge and resource centers outside the specialist health care services (9);*
- *Set up a health research and innovation council [...] to address strategic issues relating to research and innovation in the health field, develop the detailed priorities and criteria of the Ministry for financing research and innovation, develop proposals for horizontal R&D and innovation policy, [...] and process research and innovation proposals received by the Ministry (7);*
- *Establish a monitoring system for health and care research and innovation, […] assigned to the Research Council, [to] provide an ongoing and expedient knowledge base for resource use, results, and effects of research and innovation in health and care, and has a long-term ambition of encompassing all the research and innovation contributors (9);*
- *Organize deliberative fora [for the translation of research findings] (5);{, 2010, Plan d’action 2010-2013 de mise en œuvre de l’initiative EVIPNet au Burkina Faso : Amélioration de l’utilisation des résultats de recherche pour la santé. [Action Plan of Putting the EVIPNet Initiative in Place in Burkina Faso: Improving the Use of Research Results for Health.]}*
- *Establish a central arena and meeting place for monitoring the follow-up of the [KT] Strategy (9);*
- *Establish an annual health innovation forum as a networking and inspiration event for participants in the health innovation ecosystem (7);*
- *Launch a forum between researcher and policy makers (14);*
- *Launch a strategic initiative, with competitively awarded long-term (five-year) grants, based on the "networks for health innovation" concept, characterized by:*
  - *Co-governance by users (e.g., practitioners, program managers, policy-makers, the public and the private sector) and researchers, including those with expertise in KT;*
  - *Inherent flexibility with respect to precise focus (i.e. can shift focus if users' needs or science/ technology changes); and*
  - *Assessment of global scientific knowledge on the focus-question(s). (2)*
- *Provide advice on and co-fund, in collaboration with Institutes and other external sponsors, existing or planned Strategic Initiatives that build or strengthen KT networks and maximize KT potential, including partnering for scaling-up initiatives (2).*

**Box 8: Examples of strategic objectives about cooperation**

Cooperation among different institutions is also mentioned by twelve documents (1, 2, 4, 6, 9-11, 13-15, 17, 18). Examples of entities involved in such cooperation may be the following:

- *Policy-makers, planners and managers, throughout the health care, public health, and healthy public policy systems (2, 10);*
- *Education and research actors, within and across research disciplines (2, 10, 13);*
- *Health Research Board (1), Research Council (9);*
- *Patients, their families and their associations (9);*
- *National institutes of health research (4);*
- *Private sector, business, venture capital firms, manufacturers and distributors (involved in research, healthcare, health supplies, etc.) (2);*
- *Municipalities and health authorities (9, 14);*
- *Health care providers in formal and informal systems of care (2);*
- *Nongovernmental organizations (2);*
- *The general public and those who help to shape their views and/or represent their interests including the media, educators, etc. (2).*

**Box 9: Examples of strategic objectives about communication (advocacy)**

Ten documents mention activities related to communication (2, 4, 5, 7, 10, 12-14, 16, 17). These may include the following:

- *Maximize awareness of [KT-related] research, information systems and evidence reviews (16);*
- *Advocate the use of research in decisions (5);*
- *Increase the visibility of [research] projects in the wider public domain: project websites, social media presence, morning TV show appearances, YouTube, online news items, articles in print media, etc. (10);*
- *Publish annual reviews of research, translation and innovation activities (4);*
- *Develop and make available illustrative case examples of KT (2);*
- *Produce and distribute brochures and posters (14);*
- *Conduct awareness campaigns to health professionals and the general public (14);*
- *Develop mass media programs (14);*
- *Produce documentaries on [KT] activities (14);*
- *Develop and implement a KT recognition and reward initiative, e.g., provide an annual prestigious financial award for excellence in KT (2, 7);*
- *[Organize an] event of recognition and awareness-raising workshops to the impulse of activities of [KT] (7)*
- *Maintain a dialogue on public health research, organizing, participating in and contributing to discussion fora nationally and internationally (4);*
- *Build and sustain strong relationships with all stakeholders to enhance engagement and effect positive change in relation to health research (16);*
- *Conduct advocacy of [KT] activities at different fora (14);{, , National Institute for Medical Research of Tanzania. Research for Better Health! Strategic Plan 2008-2013}*
- *Enhance the [the knowledge broker’s] reputation as a leader in the provision of relevant, high-quality research, data and evidence (16).*

# *Closing part*

The closing part of KT strategies is made up of a set of different topics. Most important of those are provisions on implementation, measuring and evaluation. In some countries, research to be conducted on KT mechanisms themselves also figures in the KT strategy. Finally, a set on annexes may comprise different topics that are part of the strategy, but were long to be put in the main text.

## Implementation

Eight documents contain a separate section discussing implementation issues: responsible organizations, timelines, budgeting, measurement and evaluation, etc. (2, 5, 7, 12, 14-16, 18){, 2011, Ministry of Higher Education of Malaysia. Knowledge Transfer Programme – KTP}. This information, or pats of it, may be put directly into the tables on strategic objectives (above), but it may also make up for a distinct section towards the end of the strategy document.

## Measuring & evaluation

Measuring and evaluation (M&E) is an essential part of most strategies. Indicators may figure in the tables of objectives and actions (above), but is often dedicated a separate section (2, 4-9, 11-13, 15, 16, 18).^[[4]](#footnote-4)^ Box 10 presents some examples of topics from those M&E sections of KT strategies reviewed.

**Box 10: Examples of M&E objectives from the KT strategies reviewed**

- *Generate instruments (study and diagnostics) to measure the social impact of the research and innovation projects (12);*
- *[Establish a] KT evaluation framework [that] will guide the evaluation of the KT function and will be based on the strategic outcomes (2);*
- *Lay the basis for the regular monitoring and analysis of health research and innovation so as to be able to evaluate the results of actions and their impact on the health system and give the decision-makers a relevant overview of the situation in health research and innovation, the progress of implementation of the strategy, etc. (7);*
- *[Define] knowledge [translation] indicators, including a possible composite indicator that reflects the broad range of knowledge transfer activities (18);*
- *Continuously review progress in implementing the strategy; manage its operational delivery and monitor its on-going appropriateness; amend it as necessary in response to changes in the external environment (16);*
- *Elaborate a study on the implementation of the action plan (5);*
- *Convene an evaluation conference at mid-term [of any program] (5);*
- *Establish a board to review progress annually, as well as an interior measurement group (4);*
- *Provide information on the extent to which the actors’ implementation of the strategy is a positive development (9);*
- *Support informed expenditure decisions on research and information investments (16);*
- *Utilize financial information streams, evaluation metrics, value for money initiatives and other relevant data to ensure informed decision-making (16);*
- *[Continuously] integrate national initiatives/changes in corporate governance practices into [KT] processes and policies (16).*

##

## Research on KT

Beyond the translation of research findings to policy, the development of research into KT itself may also be an objective. Three documents (2, 11, 12)^[[5]](#footnote-5)^ mention this topic. In (2), the following interventions are proposed:

Fund grants, in collaboration with Institutes and through strategic and open competitions that support KT research in:

- Basic science of KT – theoretical and conceptual research on KT;
- KT intervention development, scaling-up and sustainability research – pilot studies and intervention strategies, including quantitative and economic models that predict the best return on research investment;
- Evaluation research – testing of evidence-based interventions or KT strategies for particular user communities or settings; and
- Knowledge synthesis – methods for gathering and synthesizing evidence for particular user audiences that respond to clear health or health system priorities.

Develop KT research capacity through:

- Funding open awards or supporting Institute-based or cross-Institutes strategic initiatives, e.g., training and personnel awards; and
- Supporting interaction among KT researchers through workshops and fora.

##

## Annexes

## Seven documents contain annexes (2, 4, 7, 8, 11, 13, 14)^[[6]](#footnote-6)^. These include the following elements:

- References, abbreviations, glossary, bibliography (2, 13);
- A list of research units [concerned by the strategy] (4);
- Strategy preparation process (7);
- Summary of comments provided through public consultation [of the strategy] (4, 14);
- [Composition of the] Knowledge Translation Working Group (2);
- [A section on] developments with the greatest impact on the future of the health system in the [strategy’s] time frame (7, 13).

# References

1. Department of Health of the Republic of Ireland. Department of Health Statement of Strategy 2016-2019. Dublin.

2. Canadian Institutes of Health Research. Knowledge Translation Strategy 2004-2009. Ottawa2004.

3. World report on knowledge for better health: strengthening health systems. Geneva: World Health Organization; 2004.

4. Doing, supporting and using public health research. The Public Health England strategy for research, translation and innovation. London: Public Health England; 2015.

5. Plan d’action 2010-2013 de mise en œuvre de l’initiative EVIPNet au Burkina Faso : Amélioration de l’utilisation des résultats de recherche pour la santé. [Action Plan of Putting the EVIPNet Initiative in Place in Burkina Faso: Improving the Use of Research Results for Health.]. Ouagadougou: Ministry of Health of Burkina Faso; 2010.

6. Ministry of Health of Jordan. Health Systems Research Strategy 2011-2015. Amman2011.

7. Research and Innovation for Health. Research, Development and Innovation Strategy for the Estonian Health System 2015–2020. Tallinn: Ministry of Social Affairs; 2015.

8. Advancing Health Research, Enhancing Life. National Institute for Medical Research Strategic Plan IV. 2014-19. Dar es Salaam: Tanzania National Institute for MEdical Research.

9. The Government Action Plan for Implementation of the Health&Care21 Strategy Action Plan Research and innovation in health and care (2015-2018). Oslo: Norwegian Ministries.

10. The University of the West Indies. Mobilizing Knowledge, Catalyzing Impact. Saint Augustine2015.

11. Canadian Institutes of Health Research. Health Research Roadmap II: Capturing Innovation to Produce Better Health and Health Care for Canadians. Strategic Plan 2014-15 - 2018-19. Ottawa2015.

12. Estrategia Nacional De Apropiacion Social De La Ciencia, La Tecnologia Y La Innovacion [National Strategy for the Social Uptake of Science, Technology and Innovation]. Bogota: Administrative Department of Science, Technology and Innovation; 2010.

13. Programa Especial de Transferencia Tecnológica 2016-2021. Versión preliminar para consulta pública. [Special Technology Transfer Program 2016-2021. Preliminary version for public consultation.]. Lima: National Council of Science, Technology and Technological Innovation.

14. National Institute for Medical Research of Tanzania. Research for Better Health! Strategic Plan 2008-2013. Dar es Salaam.

15. Ministry of Higher Education of Malaysia. Knowledge Transfer Programme – KTP. Kuala Lumpur2011.

16. Health Research Board of the Republic of Ireland. Research. Evidence. Action. HRB Strategy 2016-2020. Dublin.

17. Ministry of Health of the Kingdom of Bahrein. Health Improvement Strategy 2015-2018. Manama.

18. Norwegian ERA Roadmap 2016-2020. Oslo: Norwegian Ministry of Education and Research.

1. Out of these, two are from the same institution (the National Institute for Medical Research of Tanzania), with different time frames. [↑](#footnote-ref-1)
2. Out of these, two are from the same institution (the National Institute for Medical Research of Tanzania), with different time frames. [↑](#footnote-ref-2)
3. Out of these, two are from the same institution (the National Institute for Medical Research of Tanzania), with different time frames. [↑](#footnote-ref-3)
4. Out of these, two are from the same institution (the Canadian Isntitutes for Health Research), with different time frames. [↑](#footnote-ref-4)
5. Out of these, two are from the same institution (the Canadian Isntitutes for Health Research), with different time frames. [↑](#footnote-ref-5)
6. Out of these, four are from two institutions (the National Institute for Medical Research of Tanzania and Canadian Institutes for Health Research), with different time frames. [↑](#footnote-ref-6)
